# Preclinical study of gasless laparoscopy for radical nephrectomies in canine cadavers

**DOI:** 10.29374/2527-2179.bjvm008625

**Published:** 2026-02-02

**Authors:** Vanessa Milech, Bernardo Nascimento Antunes, Pâmela Caye, Hellen Fialho Hartmann, Marcella Teixeira Linhares, Vinicius da Silva Cadiñanos, Maurício Veloso Brun

**Affiliations:** 1 Hospital Veterinário Universitário- Campus Professora Cinobelina Elvas, Universidade Federal do Piauí, Bom Jesus, PI, Brazil.; 2 Departamento de Cirurgia e Anestesiologia, Universidade Federal do Piauí, Bom Jesus, PI, Brazil.; 3 Departamento de Cirurgia da Universidade Federal do Rio Grande do Sul, Porto Alegre, RS, Brazil.; 4 Departamento de Pequenos Animais do Instituto Federal Farroupilha, Frederico Westphalen, Frederico Westphalen, RS, Brazil.; 5 Departamento de Pequenos Animais, Universidade Regional do Noroeste do Estado do Rio grande do Sul, Ijuí, RS, Brazil.; 6 Departamento de Clínica de Pequenos Animais, Laboratório de Cirurgia Experimental, Universidade Federal de Santa Maria, Santa Maria, RS, Brazil.

**Keywords:** abdominal traction platform, kidney, laparoscopic surgery, pneumoperitoneum, canine, plataforma de tração abdominal, rim, cirurgia laparoscópica, pneumoperitônio, canino

## Abstract

In this study, we aimed to verify the feasibility of the multidirectional traction platform in performing gasless laparoscopic nephrectomies on dog cadavers. The cadavers were divided into two groups: those subjected to gasless laparoscopic radical nephrectomy (GCG) and those subjected to laparoscopic radical nephrectomy with pneumoperitoneum (GCP). The total surgical time, time for each stage of the procedure, and intraoperative complications were recorded. Using the Likert scale and visual analog scale (VAS), the surgeon and assistant assessed the degree of difficulty of each surgical approach. The total surgical time for nephrectomy was longer in the GCG group (p<0.01). Similarly, differences in the steps of positioning portal 2, establishing the abdominal elevation equipment, dissecting the vessels of the renal hilum, and dissecting the kidney from the fascia renal disease were also longer in the GCG group (p<0.05). A significant interaction was observed between the surgical group and the side of surgery, and the variable time to remove the kidney from the abdominal cavity (p=0.02) was longer in the GCG group. In the evaluation of the surgeon and assistant, the groups differed in all parameters, indicating the degree of difficulty of surgical approaches on a Likert scale (p<0.05). On the VAS scale, we observed a higher response in the GCG group (p<0.01). The multidirectional abdominal traction device used in this study enabled the performance of gasless laparoscopic radical nephrectomies on dog cadavers.

## Resumo

Neste estudo, objetivamos verificar a viabilidade da plataforma de tração multidirecional na realização de nefrectomias laparoscópicas sem gás em cadáveres caninos. Os cadáveres foram divididos em dois grupos: aqueles submetidos à nefrectomia radical laparoscópica sem gás (GCG) e aqueles submetidos à nefrectomia radical laparoscópica com pneumoperitônio (GCP). O tempo cirúrgico total, o tempo para cada etapa do procedimento e as complicações intraoperatórias foram registrados. Utilizando a escala de Likert e a escala visual analógica (EVA), o cirurgião e o auxiliar avaliaram o grau de dificuldade de cada abordagem cirúrgica. O tempo cirúrgico total para a nefrectomia foi maior no grupo GCG (p<0,01). Da mesma forma, as etapas de posicionamento do portal 2, instalação do equipamento de elevação abdominal, dissecção dos vasos do hilo renal e dissecção do rim da fáscia renal também apresentaram tempos maiores no grupo GCG (p<0,05). Observou-se interação significativa entre o grupo cirúrgico e o lado da cirurgia, sendo o tempo variável para remoção do rim da cavidade abdominal (p=0,02) maior no grupo GCG. Na avaliação do cirurgião e do auxiliar, os grupos diferiram em todos os parâmetros, indicando o grau de dificuldade das abordagens cirúrgicas em uma escala Likert (p<0,05). Na escala VAS, observou-se uma resposta maior no grupo GCG (p<0,01). O dispositivo de tração abdominal multidirecional utilizado neste estudo possibilitou a realização de nefrectomias radicais laparoscópicas sem insuflação de gás em cadáveres caninos.

## Introduction

Indications for radical nephrectomy include persistent infections, irreparable trauma, renomegalia, obstructive hydronephrosis, neoplasms (Tillson & Tobias, 2012) and unilateral renal parasitism by *D. renale* (Ferreira et al., 2010), with renal loss. Partial nephrectomy is rarely performed in small animals, and it is indicated only in cases of trauma or neoplasms located distant from the renal hilum (Brun et al., 2015).

Since laparoscopic nephrectomy became feasible, potential hemodynamic and functional insults to the kidneys have been of concern. In this context, gasless laparoscopy provides an attractive alternative to insufflation (Chiu et al., 1996). Gasless laparoscopy reduces the negative effects on the diaphragm and large abdominal vessels, as it prevents the increase in intra-abdominal pressure (IAP) and produces less pre- and post-load and cardiorespiratory effects (Casati et al., 1997; Fransson & Ragle, 2011), leading to better surgical safety in older patients or those with cardiovascular and respiratory diseases (Li et al., 2014).

Kihara et al. (2015) reported a series of 207 partial nephrectomies and 148 total nephrectomies performed using the gasless approach in human patients with peripheral renal tumors, demonstrating that it is a minimally invasive, viable, and safe technique capable of producing maximum preservation of renal function. Similarly, Watanabe et al. (2002) evaluated the effectiveness and feasibility of the abdominal lift laparoscopy technique for living donor nephrectomies, achieving success with this approach and immediate organ function after grafting. To date, there are no reports of radical nephrectomy performed in dogs using the gasless laparoscopic technique.

Notably, the changes caused by IAP, observed in renal function and perfusion, may be reversible and of little significance in healthy patients (Hazebroek et al., 2002; Hazebroek et al., 2003). However, it may also have a significant impact on patients with compromised renal function (De Seigneux et al., 2011; Dolkart et al., 2014). As laparoscopic surgery expands, subtle kidney injuries become clinically evident, especially in patients with a history of kidney injury. This condition justifies future research involving the gasless modality in animals with relevant comorbidities, such as in patients requiring unilateral radical nephrectomy.

Therefore, in this preclinical study, we aimed to evaluate the feasibility of the multidirectional traction platform for elevating the abdominal cavity during gasless laparoscopic nephrectomies in an *ex vivo* model. We hypothesized that the proposed technique would be feasible, despite potential limitations in workspace and visualization of the structures.

## Materials and methods

### Study model

The canine cadavers used came from the routine at the Santa Maria University Veterinary Hospital (HVU – UFSM), intended for study and/or research. The reasons for death or euthanasia were unrelated to the presence of nephropathies. Twenty dogs of both sexes, weighing between 5 and 16 kg, were selected. We excluded dogs with traumatic injuries to the abdominal wall or organs, recent abdominal surgical procedures, peritonitis or ascites, and the presence of abdominal changes of another nature that could interfere with the performance of the proposed surgical procedure.

The cadavers were distributed into two groups using a simple random draw. Dogs in the GCG (n=10) group underwent radical nephrectomy using the multidirectional traction platform for video surgery, and those in the GCP (n=10) group underwent radical nephrectomy with medical CO_2_ hyperbaric pneumoperitoneum. The procedures were performed randomly on both kidneys; thus, 40 nephrectomies were performed. The cadavers used were mostly frozen, without conservation, and subjected to a thawing period of 24–48 h at room temperature before the surgical procedure. The surgeries were performed by a consolidated surgical team that was still in the initial learning curve for the proposed procedures. The team was composed of a surgeon, a cameraman, an instrument nurse, and two assistants, who maintained their designated roles throughout the study.

Before performing the nephrectomies, the animals used in this study had previously been subjected to another surgical technique as part of another experimental study (Milech et al., 2023). However, to avoid interference between studies, the surgical times for each technique and the difficulties of each of them were noted and accounted for separately.

### Multidirectional abdominal lift platform

**Figure 1:**
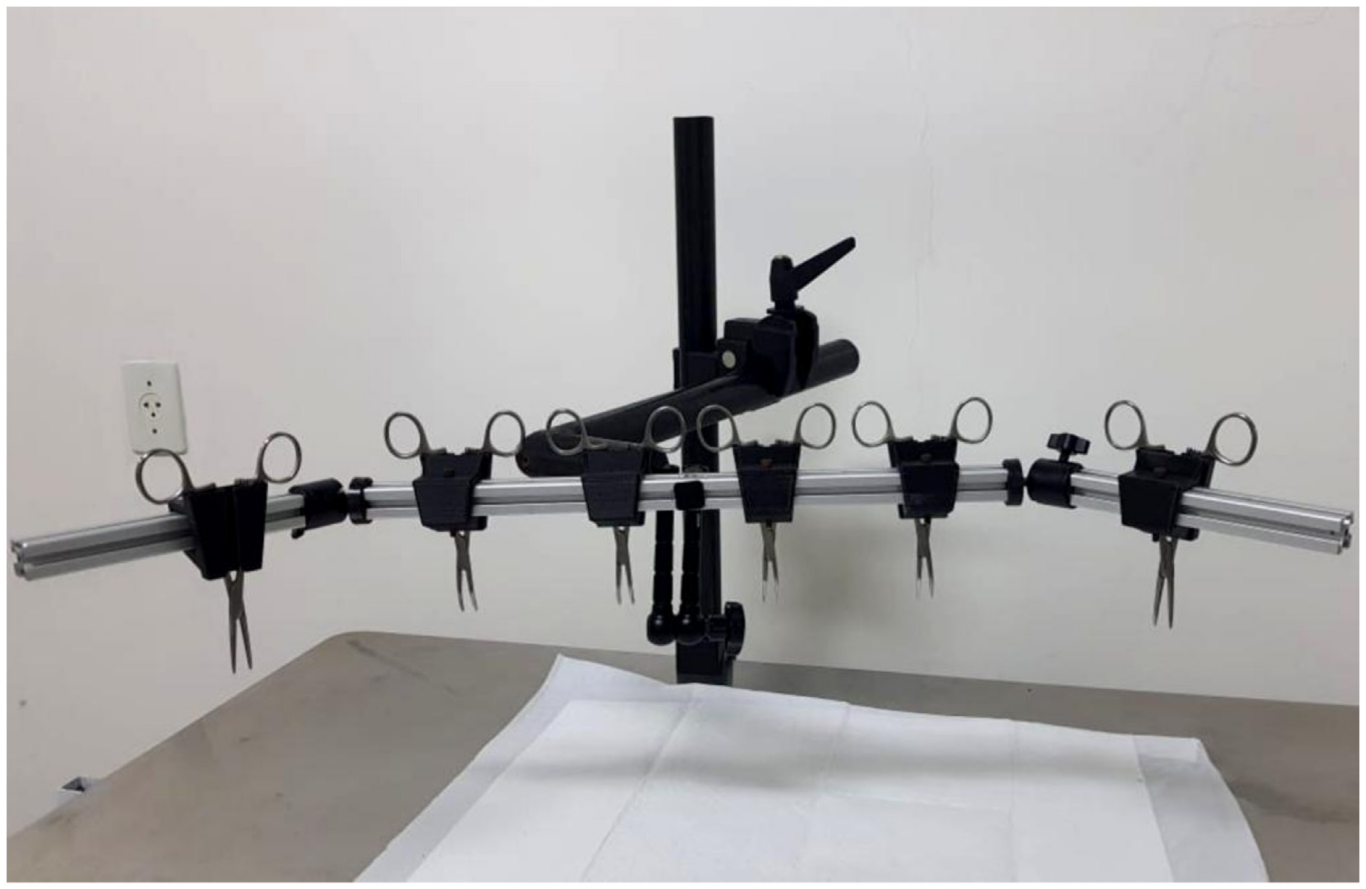
Image of the multidirectional traction platform for video surgery (ES201800465 U) (BR 102019013473-9 A2), positioned on the surgical table.

The device designed for abdominal elevation was jointly developed by UFSM, the Centro de Cirugía de Mínima Invasión Jesús Usón (CCMIJU), and the National Council for Scientific and Technological Development (CNPq). This device is registered with the *Oficina Española de Patentes y Marcas* (ES201800465 U) and at the National Institute of Industrial Property (BR 102019013473-9 A2). This abdominal traction platform was described by Brun et al. (2022) and allows the necessary adaptation for use in animals and across different conformations, anatomical spaces, and surgical indications. The device has three main parts (Figure 1), providing considerable freedom for positioning the transparietal sutures on the patient. In this way, a wide range of movements can be provided to create an intracorporeal workspace.

### Surgical procedure

#### Laparoscopic radical nephrectomy with hyperbaric pneumoperitoneum

All cadavers underwent a wide trichotomy of the right and left lateral abdominal flanks. When performing right radical nephrectomy, the canines were placed in left lateral decubitus position, with compresses wrapped under the left flank to elevate it and facilitate exposure and isolation of the contralateral kidney. For the left radical nephrectomy, the animals were placed in the right lateral decubitus position, using the same flank elevation method.

**Figure 2:**
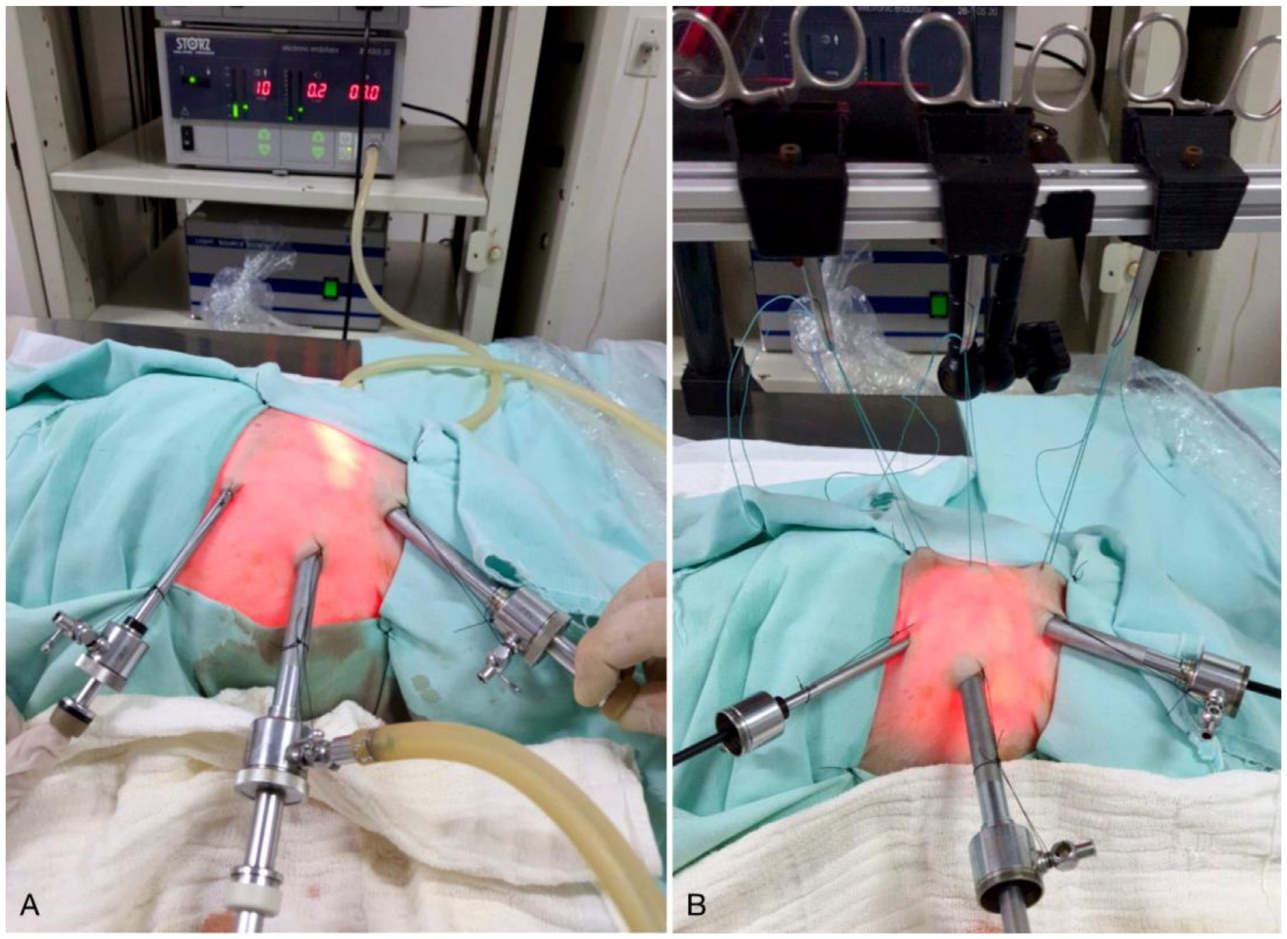
Intraoperative image of the positioning of the three portals in a triangular arrangement, to perform laparoscopic radical nephrectomy in the GCP group (A); Sutures individually fixed to conventional Kelly or Halstead hemostatic forceps demonstrating the effect of elevating the abdominal wall in the GCG group, from three anchoring points (B).

Following the methodology described by Brun et al. (2015), laparoscopic radical nephrectomy using transperitoneal access requires the use of three portals in triangulation. Using the open technique, the first 11 mm cannula (Karl Storz, Tuttlingen, Germany) was positioned on the right or left lateral abdominal wall between the iliac tuberosity and the costal margin, generally parallel to the third breast and slightly lateral to the height of the inguinal fold. Through this first portal, a 10 mm 0° rigid endoscope (Karl Storz, Tuttlingen, Germany) was passed, and the abdominal cavity was insufflated with medicinal CO_2_ at a pressure of 10 mmHg and a speed of 1.5 L/ min, using an automatic inflator (Karl Storz, Tuttlingen, Germany). Two other trocars (11 mm and 6 mm) (Karl Storz, Tuttlingen, Germany) were inserted slightly dorsal to the right and left of the first portal, respectively, forming a triangular arrangement between the portals (Figure 2A).

With the aid of a 5 mm videolaparoscopic Kelly forceps (Karl Storz, Tuttlingen, Germany) and Mixter forceps (Edlo/Exatech, Canoas, Rio Grande do Sul, Brazil), blunt dissection and isolation of the vessels of the renal hilum were performed, followed by the use of a clipper 10 mm surgical tube (Edlo/Exatech, Canoas, Rio Grande do Sul, Brazil), allowing the application of three medium-wide titanium clips (LT 300) for hemostasis of each vessel. After occlusion of the artery and vein, the vessels were sectioned between the two distal clips. The kidneys were isolated from the medial renal fascia via blunt dissection. The ureter was identified and dissected from its caudal portion and was occluded using three titanium clips close to its insertion into the urinary bladder. Next, it was sectioned between the most cranial clips. A 750 mL tissue removal bag (LapSac®, Cook Medical, Bloomington, IN, USA) was introduced into the abdominal cavity through the second 10 mm port wound. Next, the kidney was placed inside the removal bad, enabling removal of the cavity. In situations where the kidneys were enlarged, it was necessary to enlarge the access incision of the portal mentioned above. Finally, the pneumoperitoneum was undone, and the abdominal wall was sutured with polyglactin 910 2-0 (PGA 2-0, Shalon, São Luís de Montes Belo, Goiás, Brazil) in a cross-mattress pattern, followed by subcutaneous raffia in the same pattern with polyglactin 3-0 (PGA 3-0, Shalon, São Luís de Montes Belo, Goiás, Brazil). The skin synthesis was performed with 4-0 monofilament nylon (Nylon 4-0, Technofio, Goiânia, Goiás, Brazil) in a horizontal mattress pattern.

#### Gasless laparoscopic radical nephrectomy

As described for the GCP group, the GCG cadavers were also positioned according to the side on which the operation would be performed, with the appropriate elevation and angulation of the flank. Three portals with the same layout and diameter were used, as described for the GCP. The first 11 mm port was positioned using an open technique, and its correct arrangement within the peritoneal cavity was ensured by inspecting the cavity with a 10 mm 0° rigid endoscope. Next, the insertion site of the second port in the abdominal wall was determined, lateral and dorsal to the first trocar. Under direct visualization, the cannula was directed to the point chosen for inserting the second portal, thus elevating the abdominal wall and moving it away from the viscera. By partially withdrawing the endoscope into the trocar, a skin incision was made at this site and elevated by the cannula. The second trocar was inserted and directed into the first portal to avoid possible injury to the abdominal viscera. To create the intra-abdominal workspace, three transparietal support sutures were placed cranial to the already positioned portals using a No. 2 polyester thread (Polyester 2, Shalon, São Luís de Montes Belos, Goiás, Brazil). The sutures were individually fixed to conventional Halstead hemostatic forceps, previously coupled to the multidirectional abdominal traction platform to elevate the abdominal wall at the determined points (Figure 2B). Next, the sutures were pulled together at the maximum tension needed to visualize the kidneys and perform the procedure. After installing the platform, radical nephrectomy was performed in the same manner as described for the GCP group. Likewise, the occlusion of the surgical accesses was performed in three planes, after removing the transparietal suspension sutures.

### Data collection

In both groups, the surgical time was recorded for each stage from the beginning to the end of the procedure. Thus, the times recorded included the time for positioning of portals 1 (T1), 2 (T2), and 3 (T3); establishing the pneumoperitoneum or installing the platform (T4); dissecting the vessels of the renal hilum (T5); hemostasis of the renal vessels (T6); identification, occlusion and sectioning of the ureter (T7); final dissection of the kidney from the renal fascia (T8); introducing the kidney into the sac extractor and removal from the abdominal cavity (T9); to complete the synthesis of surgical accesses (T10); and finally, the total surgical time (T11).

Intraoperative complications were recorded according to severity, regarding inadvertent section of the renal and great vessels, inadequate application of titanium clips, and displacement of distal clips. and inadvertent sectioning of the ureter. Furthermore, information about weight and sex was also recorded.

To determine the degree of difficulty of the surgical approaches, both the surgeon and camera assistant completed two scales at the end of each surgical procedure. The Likert scale, based on the model presented by Tapia-Araya et al. (2015) and Keheila et al. (2017), and adapted for the surgical procedure of laparoscopic radical nephrectomy, addresses aspects of the surgical procedure from the perspective of the surgeon and assistant. Each item was classified using scores ranging from one to five (1- no difficulty, 2- low difficulty, 3- moderate difficulty, 4- high difficulty, and 5- very high difficulty). The items analyzed were: (P1) difficulty in surgical access to introduce the portals and install the multidirectional traction platform; (P2) difficulty in surgical maneuvers to perform dissection and hemostasis of the renal vessels; (P3) difficulty in surgical maneuvers to perform the dissection and removal of the kidney and ureter; (P4) difficulty in surgical maneuvers to remove the kidney from the abdominal cavity; (P5) difficulty in visualizing anatomical structures; (P6) difficulty in handling instruments considering the work space; (P7) physical fatigue; (P8) mental fatigue.

Furthermore, considering the same parameters mentioned above, the surgeon and assistant assigned a score to the visual analog scale (VAS), adapted from the scale presented by Vassiliou et al. (2005) and Chen et al. (2019), which consists of a 10 cm line with descriptive anchors at each end. The surgeon and assistant indicated with an “x” on the line, the score they assigned to indicate the technical difficulty in performing the procedure due to anatomical visualization, definition of tissue planes, instrumentation, and general technical competence. These scores were determined numerically by measuring where the “x” indication was placed along the 10 cm line. The closer it was to 0 cm, the less difficult it was to perform the corresponding step of the procedure, while values closer to 10 cm indicate greater difficulty. Regarding physical (P7) or mental (P8) fatigue experienced during the procedure, scores close to 0 cm and 10 cm indicated minimum and maximum levels of fatigue, respectively.

### Statistical analysis

Data on the surgical time steps and the surgical difficulty scale (VAS) in cm were tested for normality (PROC UNIVARIATE; SAS^®^) using the *Kolmogorov-Smirnov* test. Differences between surgical groups and between the sides of surgery for these responses were analyzed using the MIXED procedure (SAS^®^ version 9.4, SAS Institute, Cary, NC). The Tukey-tuned *lsmeans* feature compared means. The statistical model for this stage of analysis included the surgical groups (n = 2, GCG and GCP), the side of surgery (n = 2, left and right), and their interactions as fixed effects. Dogs (n = 20) and error were included as random effects. The sex and weight of the animals were included in the model as covariates. With the exception of the synthesis variable, all other surgical time variables and the VAS scale were transformed to achieve normality. Given that the degree of difficulty of surgical approaches are classificatory variables, they were analyzed using the GLIMMIX procedure of SAS^®^, Studio version, with the means compared using the *lsmeans resource* adjusted for *Tukey*. The evaluators (surgeon and assistant) of the degree of difficulty of the surgical approaches were included in the model due to their lack of symmetrical responses (Kendall correlation). For these variables, the statistical analysis model included the surgical groups (n = 2, GCG and GCP), the side of surgery (n = 2, left and right), the evaluator (n = 2, surgeon and assistant), and the interaction between group and side as fixed effects. Dogs (n = 20) and error were analyzed as random effects. The frequency of complications during surgery was calculated using the FREQ procedure. The probability of complications occurring was calculated using the GLIMMIX procedure of SAS^®^, Studio version. Differences with a p-value < 0.05 were considered statistically significant.

## Results

The canines used in the study had an average body weight of 8.26 ± 0.69 kg and were mostly female (55%). Regarding intraoperative complications, cases of inadvertent sectioning of the renal and great vessels, displacement of the distal clips of the ureter and renal vessels, and inadvertent sectioning of the ureter were recorded. The occurrence of complications during surgery was similar among the techniques evaluated (gasless or pneumoperitoneum) (p>0.05; Table 1). We observed that 30.0% of the animals had at least one complication during GCG, whereas this value was 22.5% with GCP (Table 1).

**Table 1:** Frequency and probability of occurrence of surgical complications related to gasless laparoscopic radical nephrectomy, or with pneumoperitoneum, in canine cadavers.

Surgical complications, n ( %)	Group^1^		EPM^2^	P*
	GCG	GCP		
Displacement of the distal ureter clips	6 (15.0)	2 (5.0)	0.09	0.12
Displacement of the distal clips of the renal vessels	3 (7.5)	3 (7.5)	0.08	0.99
Inadvertent section of the ureter	1 (2.5)	1 (2.5)	0.05	0.99
Inadvertent sectioning of large vessels	2 (5.0)	3 (7.5)	0.07	0.64
Totals	12 (30.0)	9 (22.5)	0.11	0.35

1 Group: GCG = Gasless laparoscopic nephrectomy; GCP = Laparoscopic nephrectomy with pneumoperitoneum.

2 EPM = Standard error of the mean;

* Probability of occurrence using the Glimmix test at 5%.

**Figure 3:**
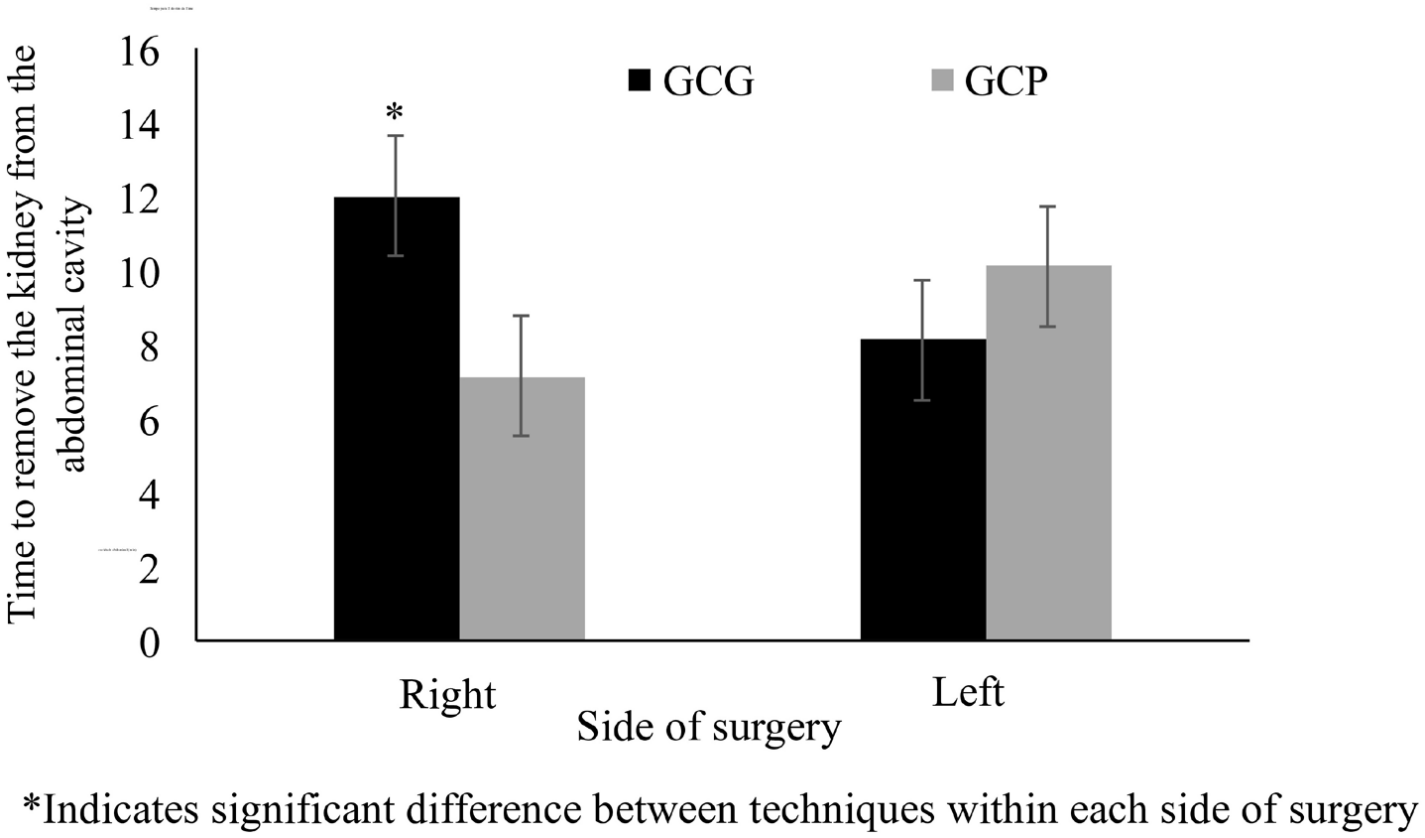
Time to remove the kidney from the abdominal cavity in relation to the surgical groups of gasless laparoscopic nephrectomy (GCG) and hyperbaric pneumoperitoneum with CO_2_ (GCP) and the side of surgery.

The total surgical time for nephrectomy was 21.83 min longer when GCG was used (p<0.05: Table 2). Regarding the time required for performing each stage of the nephrectomy, we observed differences in the time required for positioning of portal 2 (T2), establishing the abdominal elevation equipment (T4), dissecting the vessels of the renal hilum (T5) and dissecting the kidney from the fascia renal (T8), all of which were longer in the GCG group (p<0.05; Table 2). The side of surgery did not influence the total surgical time and the time for each stage when performing the nephrectomy (p>0.05; Table 2). However, we observed a significant interaction between the surgical group and the side of surgery for the time taken to remove the kidney from the abdominal cavity (T9) (p<0.05; Table 2). The time taken to remove the right kidney was longer in the GCG group than in the GCP group (p<0.05; Figure 2). For the time taken to remove the left kidney, no difference was observed between the groups (p>0.05; Figure 3).

**Table 2:** Total surgical time and stages of surgical time for nephrectomy according to the surgical groups evaluated and the side of surgery.

Surgical times (min)	Group (G)^1^		Side (L)		EPM^2^	Odds^3^		
	GCG	GCP	Right	Left		G	L	G*L
Portal positioning 1	3.18	2.70	2.91	2.97	0.26	0.28	0.94	0.44
Portal positioning 2	2.25a	1.46b	1.87	1.84	0.15	**<0.01**	0.88	0.60
Portal positioning 3	2.11	1.72	1.87	1.96	0.19	0.15	0.78	0.45
Establishment of lifting equipment	1.63a	0.42b	1.23	0.82	0.16	**<0.01**	0.05	0.65
Dissection of the vessels of the renal hilum	9.37a	5.76b	7.35	7.78	0.88	**0.01**	0.45	0.83
Hemostasis of renal vessels	7.09	4.45	5.00	6.54	1.04	0.24	0.79	0.17
Identification and section of the ureter	11.06	6.04	7.73	9.38	1.58	0.07	0.48	0.87
Dissection of the kidney from the renal fascia	7.03a	4.92b	6.18	5.77	0.68	**0.02**	0.48	0.35
Removal of the kidney from the abdominal cavity	10.06	8.62	9.56	9.11	1.09	0.48	0.92	**0.02**
Conclusion of the synthesis of surgical accesses	7.07	7.16	7.06	7.17	0.31	0.85	0.79	0.52
Total surgical time	73.32a	51.49b	62.88	61.92	4.41	**<0.01**	0.86	0.34

1 Group: GCG = Gasless laparoscopic nephrectomy; GCP = Laparoscopic nephrectomy with pneumoperitoneum;

2 EPM = Standard error of the mean;

3 Probability by Tukey test at 5%.

Regarding the evaluation of the surgeon and assistant, no significant interactions were observed between the surgical group evaluated and the side of surgery for the variables related to the degree of difficulty of the surgical approaches on the Likert and VAS scales (p<0.05; Table 3). However, the groups differed in relation to almost all parameters, indicating the degree of difficulty of surgical approaches on a Likert scale (p<0.05; Table 3). With the exception of mental fatigue (P8), which was similar between groups (p>0.05; Table 3), all other parameters showed a higher score for the GCG technique. For the VAS scale, we also observed a higher response in the GCG group (p<0.05; Table 3). The side of surgery did not influence the parameters indicating the degree of difficulty of surgical approaches on the Likert and VAS scales (p>0.05; Table 3).

**Table 3:** Degree of difficulty of surgical approaches during nephrectomy on the Likert and VAS scales, according to the surgical groups evaluated and the side of surgery.

Parameters^2^	Group (G)^1^		Side (L)		EPM^4^	Odds^5^		
	GCG	GCP	Right	Left		G	L	G*L
P1	1.60a	1.27b	1.55	1.32	0.09	**0.03**	0.10	0.58
P2	2.99a	2.11b	2.52	2.52	0.13	**<0.01**	0.99	0.40
P3	2.97a	2.13b	2.75	2.35	0.15	**0.01**	0.05	0.62
P4	2.99a	2.36b	2.65	2.70	0.14	**0.01**	0.80	0.80
P5	2.75a	1.64b	2.32	2.07	0.13	**<0.01**	0.14	0.55
P6	2.84a	1.86b	2.47	2.22	0.13	**<0.01**	0.18	0.99
P7	1.95a	1.62b	1.75	1.82	0.10	**0.04**	0.61	0.86
P8	1.67	1.45	1.50	1.62	0.09	0.14	0.35	0.85
EVA (cm)^3^	4.42a	2.41b	3.55	3.28	0.26	**<0.01**	0.33	0.93

1 Group: GCG = Gasless laparoscopic nephrectomy; GCP = Laparoscopic nephrectomy with pneumoperitoneum;

2 Likert scale parameters: P1= difficulty in surgical access to introduce portals and install the multidirectional traction platform; P2= difficulty in surgical maneuvers to perform dissection and hemostasis of the renal vessels; P3= difficulty in surgical maneuvers to perform the dissection and removal of the kidney and ureter; P4= difficulty in surgical maneuvers to remove the kidney from the abdominal cavity; P5= difficulty visualizing anatomical structures; P6= difficulty in handling instruments considering the work space; P7= physical fatigue; P8= mental fatigue;

3 VAS surgical difficulty scale;

4 EPM = Standard error of the mean;

5 Probability by Tukey test at 5%.

## Discussion

The authors believed that, as these were cadavers, it would be prudent to perform the surgical technique on both kidneys (right and left). In this way, a simple random draw was performed for the surgical group (GCG or GCP) and subsequently for the first side to be operated on, thus avoiding the surgeon's tendency to repeatedly and sequentially perform the surgical steps.

Performing laparoscopic radical nephrectomies in the gasless modality, using the multidirectional abdominal traction device, proved to be effective in cadavers. The procedures were associated with longer surgical times, difficulties in visualizing surgical maneuvers, and some surgical complications; however, conversion to conventional laparoscopy was not required.

The rates of intraoperative complications were higher than those reported in other studies that evaluated laparoscopic nephrectomies (Hayakawa et al., 2004; Kim et al., 2013; Mayhew et al., 2013), especially in the GCG group. Displacement of the distal clips (removed with the organ), both from the ureter and the renal vessels, is largely due to excessive manipulation of the organ during dissection and during its bagging for removal from the abdominal cavity. In the GCG group, the reduced working space can be considered a major cofactor for the displacement of the clips. However, we must also consider that the titanium metal clips have some disadvantages, including sliding, the low tissue retention capacity, and the possibility of incomplete engulfment of the renal vein (Kim et al., 2013; Ping et al., 2010). Another aspect is that during the transection of the vessels, at least 1–2 mm of the renal artery and vein distal to the clip must be maintained to avoid clip slippage and blood leakage (Ping et al., 2010). However, we could not ensure this in all canines in the GCG group due to the lack of working space.

Regarding other complications, Mayhew et al. (2013) reported a case of incomplete resection of the ureter due to difficulty in visualization, even during insufflation of the abdominal cavity with CO_2_. In the cases in this study, we experienced the greatest difficulty during the visualization of the structures, which culminated in the inadvertent sectioning of the ureter. However, as there was one case in each group, we also attribute this difficulty to the reduced differentiation of the tissues in the *ex vivo* model, inherent to *post-mortem* changes, which partially hindered the achievement of the appropriate dissection plane, contributing to the occurrence of this complication. Furthermore, inadvertent sectioning of large vessels occurred in the renal artery and vein, as well as in the external iliac artery, and during dissection of the ureter close to the urinary bladder. There was a greater tendency for inadvertent sectioning of additional renal arteries, as most kidneys receive irrigation from a single artery; however, multiple renal arteries are reportedly seen in 13% of canine kidneys (Tillson & Tobias, 2012). We observed that, while attempting to follow the surgical technique described *in vivo*—which initially indicates ligating the artery before the renal vein, to prevent the accumulation of blood and provide a greater degree of hemostasis (Hayakawa et al., 2004)—we failed to accurately identify another arterial branch or a double renal artery due to the difficulty in differentiating due to the appearance and color of the structures in the cadaver. Mayhew et al. (2013) and Ping et al. (2010) reported that the stages of dissection, ligation, and transection of the renal vascular pedicle are crucial in laparoscopic nephrectomy, and that potential complications must be anticipated and avoided. In our study, we believe that the high number of complications was associated with the team's learning curve and the difficulty in identifying the anatomical structures in the proposed model.

The gasless laparoscopic technique was performed in an inadequate operating space (Wu et al., 2014), and this was quite visible as we repeatedly observed greater difficulty in placing the right kidney inside the extraction bag and removing it from the abdominal cavity for cadavers in the GCG group. This difference may be due to the anatomical positioning of this organ, which is found more cranially in the abdominal cavity (Tillson & Tobias, 2012; Fossum, 2007; Evans & De Lahunta, 2013), making its traction towards the caudal and lateral segments of the cavity, as well as its insertion into the sac extractor, more laborious. In addition, when the extractor bag was placed in the cavity on the right side, it entered in a cranio-caudal direction, remaining in the caudal portion of the abdomen and between different segments of the intestine, making it difficult to open, which can be even more challenging in confined workspaces. When it was inserted on the left side, it entered in the caudo-cranial direction, remaining in the cranial portion of the abdomen, in a location with more compact organs (liver and spleen) and without the mesentery, facilitating its opening and positioning of the kidney in its interior.

Furthermore, we chose to remove the entire organ from the bag for tissue removal, without performing morcellation, and when necessary, enlarge the surgical wound in the second portal by approximately 8 mm, as we believed that this would require less surgical time. The scientific literature does not establish a clear consensus regarding the extraction of renal specimens from the intra-abdominal space; however, Secchi et al. (2010) and Mayhew et al. (2013) removed the kidney after placing it in a tissue extraction bag through the surgical wound in the second portal, enlarging the incision proportionally to the size of the organ. In contrast, in a study carried out by Kim et al. (2013), the kidney was extracted by morcellation, avoiding contamination of the wound. However, the authors reported that this procedure took more time than removing the intact specimen, but with the surgeon's experience, it could be performed very quickly. Other authors (Brun et al., 2015) indicated reduced enlargement of the access wound and fragmentation of the bagged specimen, as they believe that, despite taking more time, the absence of extensive enlargement considerably reduces tissue damage.

The mean surgical time for laparoscopic radical nephrectomy was 73.32 min (±4.41) and 51.49 min (±4.41) in the GCG and GCP groups, respectively, with the time being significantly longer in the GCG group. This can be attributed to the more critical surgical steps in the procedure, such as the positioning of the second portal, establishment of the abdominal lifting equipment, dissection of the renal hilum vessels, and dissection of the kidney from the renal fascia. In the literature, we found surgical times ranging from 51.9 min (Kim et al., 2013) to 80 min (Mayhew et al., 2013) to perform conventional laparoscopic nephrectomies in dogs. In line with the meta-analysis by Gurusamy et al. (2018), the surgical time was also longer in human patients undergoing laparoscopy with elevation, associated in part with poorer exposure of the abdominal cavity. In addition, the authors state that, in most of the studies analyzed, an additional average time interval of 3–24 min was observed in gasless laparoscopic cholecystectomies but not in conventional cholecystectomies. Similarly, in their systematic review, Aruparayil et al. (2021) demonstrated that operative times for gasless general and gynecological surgeries were longer than those for abdominal insufflation laparoscopy. In contrast, Hayakawa et al. (2004), evaluated the performance of laparoscopic nephrectomies in humans, and the operation time tended to be shorter in the gasless group. Furthermore, Kim et al. (2020) found no difference in surgical times for performing gasless laparoscopic hysterectomies and suggested that the abdominal retraction system allowed for adequate configuration of the operating field to perform the procedure. Given the discrepancy in results found in the literature, performing gasless laparoscopy appears to be technically challenging and is related to the type of surgical procedure performed, the team's learning curve, and the characteristics of the lifting device applied to the surgical technique.

Using the Likert and VAS scales, we identified that the surgeon and assistant rated intracavitary surgical maneuvers as more difficult in the GCG group, consistent with the longer surgical times observed in this group. Most surgeons would agree that instruments and visual performance obstacles often make laparoscopic surgery more stressful (Berguer et al., 2001). Therefore, one of the main concerns of surgeons in adopting the gasless technique is related to the operative field of view and safety issues, including damage to the abdominal wall during elevation (Bolton et al., 2021).

Most lifting systems consist of an anchoring part inserted directly into the abdominal cavity (Fransson & Ragle, 2011; Wu et al., 2014; Kim et al., 2020; Fransson et al., 2015; Chen et al., 2011) or subcutaneously (Akira et al., 2005; Hashimoto et al., 1993; Palomba et al., 2010), and another portion for traction, fixed to a rigid pillar or support structure (Alijani & Cuschieri, 2001). The abdominal elevation device presented and used in this study allows adjustment to various anatomical conditions, by employing multiple transparietal elevation sutures placed at different locations in the abdominal region. This device effectively repaired diaphragmatic hernias in canine cadavers, providing adequate working space for cranial abdominal surgery (Brun et al., 2022). However, it has not yet been applied to surgeries in the lateral or caudal abdominal region.

Notably, many devices produce a tent effect, creating an angled cavity that can restrict vision (Bolton et al., 2021), leading to inferior exposure of the lateral aspects of the abdominal cavity (Fransson & Ragle, 2011; Kennedy et al., 2015), in contrast to the dome-shaped exposure promoted by pneumoperitoneum. The pressure caused is uniform and exerted across the entire abdominal wall, rather than concentrated as tension at focal points (Alijani & Cuschieri, 2001; Kennedy et al., 2015). In a study conducted using the aforementioned traction platform (ES201800465 U) (BR 102019013473-9 A2) for diaphragmatic herniorrhaphies (Brun et al., 2022), the tent effect did not affect the execution of the procedures, probably because the rib cage and sternum provide structural support to the abdominal wall and assist surgical exposure when approaching the cranial abdominal region (Kennedy et al., 2015).

Watkins et al. (2013) reported that CO_2_ insufflation would probably enable better observation of structures in the lateral aspects of the abdominal cavity compared to the tent shape of the gasless approach; however, this deficiency could be overcome by placing several elevation retractors and alternating placement in more lateral locations. Fransson & Ragle (2011) described that visualization can be negatively affected when elevating the dog in lateral recumbency, due to the exaggerated tent effect created on the lateral abdominal wall; however, auxiliary or additional lifting devices could improve visualization. We expected that using three traction sutures placed at different points cranial to the work portals would minimize the tent effect on the visibility of the surgical site; however, it was difficult to create a workspace on the surgical site on the side of the abdominal cavity.

Fransson et al. (2015) stated that gasless laparoscopy cannot be performed in surgeries that require broad and excellent intra-abdominal visualization, as reduced visualization can prolong the surgical procedure. In addition, Aruparayil et al. (2021) stated that gasless laparoscopy would be more suitable for simpler diagnostic procedures, cholecystectomies, and other gynecological procedures in humans. Laparoscopic radical nephrectomy is considered a complex surgery in dogs when performed with hyperbaric pneumoperitoneum at pressures of 10–12 mmHg (Mayhew et al., 2013), which explains the difficulties encountered in the present study when performing the approach within a reduced working field. We emphasized that, as the learning curve for the specific technique is overcome, the procedure tends to be less laborious and the risk of complications decreases. Monitoring to ensure that a safe traction force is applied is also essential, as elevating the abdominal wall can cause trauma if an excessive force is applied (Bolton et al., 2021). Once the caudal trunk begins to rise from the surgical table, maximum lengthening of the abdominal body wall has likely been achieved, and no gain in abdominal volume would be achieved (Kennedy et al., 2015). A study conducted in dogs using the gasless technique for various surgical procedures revealed that in many patients, the necessary tension was considered quite high, especially in particular surgical stages, such as locating the ovaries (Fransson & Ragle, 2011). To the best of our knowledge, the lifting required for optimal visualization of the different surgical approaches has not been determined. Therefore, in the present study, the lifting tension of the abdominal wall was not determined. However, considering the *in vivo* applicability, we believe that tissue trauma and discomfort caused by the lifting device must be evaluated, avoiding applying high support tension.

Pneumoperitoneum requires valved portals to maintain insufflation with CO_2_, and the size of the portals limits the instrumentation that can be effectively used (Watkins et al., 2013). Gas leaks result in temporary reductions in the working space and impair the visibility of the operative field, prolonging surgical time, considering the periods required to reinflate the cavity (Maurin et al., 2020). In this regard, abdominal elevation laparoscopy is advantageous as it allows adequate visualization even when using non-valved portals, without the need to reinflate the abdomen, and uses instruments of different sizes (Fransson & Ragle, 2011). In our study, we verified the practicality of the gasless approach*,* as it does not require valved portals during the transition of instruments, especially during dissection, hemostasis, and sectioning of the renal vessels and ureter. In our experience, permanent cannulas can be disassembled from their inflation and pneumoperitoneum maintenance valves, in addition to their sealing rubbers. This makes the cannulas much lighter, facilitates the placement and removal of surgical gauze, and even allows the use of more than one instrument simultaneously through 10 mm or larger portals (for example, the simultaneous use of 3 mm and 5 mm instruments through the same cannula).

This study has some limitations. The use of cadavers makes it impossible to evaluate several variables, such as physiological parameters, hemostasis of the renal hilum vessels, the impact of intraoperative complications, and the pain caused by abdominal traction, compared with the painful stimulus of abdominal distension and visceral compression by hyperbaric pneumoperitoneum. Furthermore, the use of frozen or refrigerated corpses, without conservation, made the stages of exposure of the renal hilum and ureter more complex owing to changes in the color of the organ. An alternative would be to use appropriate preservation and storage techniques for study models, such as glycerination methods (Maciel et al., 2020), modified Larssen solutions (Oliveira et al., 2018), or curing salts (Pereira et al., 2019; Oliveira, 2014).

Considering the difficulty in performing the procedure associated with the reduced working space caused by the gasless technique compared to performing nephrectomy via hyperbaric pneumoperitoneum, patients should be carefully selected in case of *in vivo* application. Clinicians must weigh the benefits of avoiding pneumoperitoneum hypertensive with CO_2_ versus the feasibility and safety of gasless surgery. If future *in vivo* studies demonstrate that this new proposed gasless technique is safe and viable, we hypothesize that possible candidates are patients with atrophic kidneys or those without renal organomegaly, a condition that would further limit the workspace already reduced by the nature of the gasless procedure*.*

## Conclusion

The findings of this study can be used to perform gasless laparoscopic radical nephrectomies on dog cadavers. However, considering our evaluations, which revealed that it is a more difficult surgery than the laparoscopic approach with hyperbaric pneumoperitoneum, the benefits compared to standard laparoscopic surgery should be evaluated *in vivo* before a possible clinical indication. Furthermore, considering the importance of the gasless modality for patients with nephropathies, older patients or those with cardiorespiratory comorbidities, further studies demonstrating the benefits (or not) of this new technique for dogs are justified.
